# Clinical and Structural Features of Sperm Head Vacuoles in Men Included in the In Vitro Fertilization Programme

**DOI:** 10.1155/2014/927841

**Published:** 2014-04-09

**Authors:** Nina Fekonja, Jasna Štrus, Magda Tušek Žnidarič, Katja Knez, Eda Vrtacnik Bokal, Ivan Verdenik, Irma Virant-Klun

**Affiliations:** ^1^Department of Biology, Biotechnical Faculty, University of Ljubljana, Vecna pot 111, SI-1000 Ljubljana, Slovenia; ^2^National Institute of Biology, Vecna pot 111, SI-1000 Ljubljana, Slovenia; ^3^Reproductive Unit, Department of Obstetrics and Gynecology, University Medical Centre Ljubljana, Slajmerjeva 3, SI-1000 Ljubljana, Slovenia

## Abstract

The human sperm head vacuoles and their role in male infertility are still poorly understood. The aim of this study was to identify the clinical and ultrastructural features of human sperm head vacuoles in men included in the in vitro fertilization programme: men with normal (normozoospermia) and impaired sperm morphology (teratozoospermia). The sperm samples were observed under 6000-time magnification using motile sperm organelle morphology examination (MSOME). The proportion of sperm with head vacuoles was evaluated and related to the outcome of in vitro fertilization. The sperm of men with impaired sperm morphology was characterized by a higher proportion of sperm head vacuoles. The sperm head vacuoles were related to impaired semen quality (sperm concentration, motility, and morphology) but were not influenced by male factors (semen volume, height, age, weight, or body mass index). Moreover, sperm head vacuoles were related to impaired fertilization rate merely after classical in vitro fertilization (IVF), while there was no relation to pregnancy. In a subgroup of men, the sperm was fixed and observed by transmission electron microscopy (TEM). The ultrastructural study revealed that sperm head vacuoles are large nuclear indentations of various sizes and positions, packed with membranous material organized in membrane whorls (MW).

## 1. Introduction

Male infertility can be related to abnormal morphology of spermatozoa. In the last years, there has been much debate on a new potential parameter of sperm quality and male fertility, sperm head vacuoles. In spite of that, the human sperm head vacuoles and their potential role in male infertility are still poorly understood.

Using motile sperm organelle morphology examination (MSOME), where living sperm cells are visualized with DIC Nomarski microscopy at magnification of 6000 times, some fine morphological structures are visible which might be useful to determine the quality of sperm. This method shows small and larger surface concavities termed sperm vacuoles, usually located in the head of sperm cells. The nature and origin of sperm head vacuoles are still uncertain and it is not clear whether they originate from the nucleus or acrosome or other membranous structures. Vacuoles are also found in almost all samples with normal sperm morphology (>14% morphologically normal spermatozoa according to Kruger's strict criteria) and are mainly located at the tip or middle area of sperm heads [1]. The three-dimensional deconvolution images, which detect DNA, showed vacuoles as “nuclear concavities” and atomic force microscopy revealed intact but slightly sunken plasma membrane in the area of vacuoles [2]. Moreover, the electron microscope images of sperm samples in patients with abnormal sperm morphology, teratozoospermia (<14% normal spermatozoa in the sample according to Kruger's strict criteria), showed vacuoles exclusively in the area of sperm nucleus [3]. The anterior location of sperm head vacuoles led to the question about their possible origin from acrosome. The induction of acrosome reaction by ionophore A23587 [4], follicular fluid, and hyaluronic acid [5] decreased the percentage of spermatozoa with head vacuoles which suggested that they might be related to the acrosome. However, vacuoles are also present in the sperm samples without acrosome (i.e., globozoospermia), suggesting that they do not originate from acrosome [6]. It was shown that samples with large sperm head vacuoles (more than 13% of head surface) have higher percentage of fragmented DNA compared to those with small vacuoles [7] as well as higher frequency of chromosome abnormalities [3]. The sperm head vacuoles observed under the high magnification of light microscopy were described as “pocket-like” nuclear concavities related to failure of chromatin condensation [3, 8]. On the contrary, some researchers did not find any correlation between the large sperm head vacuoles and DNA damage [1] or chromosome abnormalities [1, 2]. The sperm nucleus modelling during spermiogenesis involves nucleoproteins, microtubular manchette, and perinuclear material which is known to participate in the acrosome anchorage on the nucleus and also constitutes the perinuclear theca [9]. During spermiogenesis, the nucleus becomes strikingly reduced and the nucleosomes usually appear nonrandomly distributed throughout the genome. The nucleosome-bound DNA may stay assembled with nuclear matrix and is seen as “empty space” in electronic images. Together they could have important role in paternal chromatin structure following fertilization [10]. In the late steps of spermiogenesis, the nuclear DNA becomes highly condensed because the nucleoproteins histones are replaced by protamines [11]. During replacement of nucleoproteins, many temporarily functional proteins are formed and degraded in special degradation sites. The nuclear pockets at the base of sperm head were proposed to function as proteolytic centers and the exit site from where the protein residues leave the nucleus and may be functionally connected with vacuoles [11, 12].

The sperm head vacuoles are an interesting but poorly understood parameter of sperm quality and male fertility which seem to be related to the outcome of in vitro fertilization in men with impaired sperm quality [13–15]. The origin and dynamics of sperm head vacuoles are not well understood and their role in sperm function is disputable. Some authors suggest that sperm vacuoles are normal features of sperm head [1, 16], while others describe them as degenerative structures related to male subfertility [2, 17, 18]. Therefore, the aim of this study was to evaluate the percentages of sperm with head vacuoles in both the normal sperm samples and samples with impaired sperm morphology and to relate them to the outcome of in vitro fertilization. Moreover, the ultrastructure and function of sperm head vacuoles were reconsidered in relation to sperm maturation, using the high resolution of transmission electron microscopy.

## 2. Materials and Methods

In this study, the sperm samples of 81 men included into the in vitro fertilization programme were examined: 40 men with normal sperm morphology and 41 men with abnormal sperm morphology (teratozoospermia) according to Kruger's strict criteria (abnormal: <14% normal sperm). The sperm samples were prepared and used for in vitro fertilization. Each sperm sample was prepared by centrifugation (300 g) for 20 min on 100%/40% density gradient of PureSperm (NidaCon, Sweden) followed by the swim-up technique, where the best spermatozoa were extracted from the sample. All normal sperm samples were used for classical in vitro fertilization (IVF), while the samples with abnormal sperm morphology were used for intracytoplasmic sperm injection (ICSI) to fertilize the female partner's oocytes. The samples, which remained after in vitro fertilization, were used for motile sperm organelle morphology examination (MSOME) to evaluate the percentage of sperm head vacuoles in each sample under 6000-time magnification. The percentages of sperm with head vacuoles and the outcome of in vitro fertilization were compared between the normal and abnormal sperm samples. Using the high resolution of transmission electron microscopy (TEM), the ultrastructure of 10 samples of normozoospermic men and 6 samples of teratozoospermic men was observed. The potential function and structure of vacuoles were searched, also in relation to sperm maturation process.

This research was approved by the National Medical Ethics Committee.

### 2.1. Classical Parameters of Semen Quality

The classical parameters, sperm concentration and motility and morphology of each sperm sample, were evaluated. The sperm sample was supposed to be normal at concentration ≥15 million spermatozoa per mL and ≥40% total (progressive and nonprogressive) motile spermatozoa according to WHO criteria [19] and at ≥14% of morphologically normal spermatozoa according to Kruger's strict criteria.

### 2.2. Motile Sperm Organelle Morphology Examination (MSOME)

For motile sperm organelle morphology examination, we used droplets of SpermSlow medium (Origio, Denmark) in a glass-bottomed dish (GWSt1000; Will Co, Wells, Amsterdam, The Netherlands) and droplets of sperm sample prepared by swim-up technique, previously described by Knez [20]. The spermatozoa in each sample were monitored under an inverted microscope with a heated stage equipped with differential interference contrast (Eclipse TE2000-S; Nikon, Japan). One thin, elongated droplet of SpermSlow medium (Origio, Denmark) was placed on the bottom of a glass dish. A smaller droplet of prepared spermatozoa was placed near SpermSlow droplet and spermatozoa bound to hyaluronate. All droplets were covered with paraffin oil to prevent drying of the sperm sample. For observation under 6000 magnification, a droplet of immersion oil was inserted underneath the glass dish (under the SpermSlow droplet). A droplet of SpermSlow with bound spermatozoa was monitored by the immersion objective, differential interference contrast, and a Nikon Digital Sight DS-Ri1 camera. In each sample, 200 spermatozoa bound to the hyaluronate were chosen and evaluated according to head vacuoles, at 6000-time magnification. The scoring system for the evaluation of each spermatozoon was based on the following classification of Vanderzwalmen [14]. According to the number of vacuoles and their size, spermatozoa were classified into the following: (1) Class I: absence of vacuoles, (2) Class II: maximum of two small vacuoles, (3) Class III: more than two small vacuoles or at least one large vacuole, and (4) Class IV: large vacuoles in conjunction with abnormal head shapes or other abnormalities. In each sperm sample, the proportions of Class I, Class II, Class III, and Class IV spermatozoa were evaluated. For statistics, the proportions of Class I and Class II spermatozoa were combined into one group, “good sperm,” and the proportions of Class III and Class IV sperm into one group, “bad sperm,” in 3 terms of sperm head vacuoles.

### 2.3. Transmission Electron Microscopy (TEM)

To describe the ultrastructural characteristics of vacuoles in sperm nucleus, chosen sperm samples were centrifuged at 500 g for 10 minutes at room temperature. Supernatants were carefully removed and pellets were fixed in 2.5% glutaraldehyde and 1% paraformaldehyde in 0.1 M PB (Sörensen's phosphate buffer), pH 7.0 for 24 h at 4°C, postfixed with 1% OsO_4_ in 0.1 M PB for 1.5 h, and embedded in Agar 100 resin (Agar Scientific). Ultrathin sections were stained with 4% water solution of uranyl acetate and Reynolds lead citrate. Samples were examined with Philips CM 100 electron microscope (FEI, The Netherlands), operating at 80 kV; images were recorded with Bioscan 792 and Orius 200 camera (Gatan) using Digital Micrograph, Software (Gatan Inc., Washington, DC, USA) at magnifications from 1100 to 92000 times.

### 2.4. Statistical Analysis

The statistical significance of differences in the percentages of sperm with sperm head vacuoles and the outcome of in vitro fertilization (pregnancy rate) between the normal sperm samples and samples with abnormal morphology (teratozoospermia) were evaluated by chi-square test. The percentages of “good sperm” (Class I and Class II), “bad sperm” (Class III and Class IV), and Class I, Class II, Class III, and Class IV sperm in terms of sperm head vacuoles were correlated with classical parameters of semen quality (concentration, motility, and morphology), male factors (semen volume, leukocytospermia, age, height, weight, and body mass index), and the outcome of in vitro fertilization (fertilization, blastocyst, and pregnancy rates) using Spearman's rho correlation. For the outcome of in vitro fertilization, the data were evaluated for total in vitro fertilization cycles and separately for classical in vitro fertilization (IVF) and intracytoplasmic sperm injection (ICSI). The pregnancy dependence on female and male factors (including sperm head vacuoles) was evaluated by multivariate logistic regression. The statistical significance was set at *P* < 0.05.

## 3. Results and Discussion

### 3.1. Motile Sperm Organelle Morphology Examination (MSOME)

#### 3.1.1. Sperm Head Vacuoles as Seen by Light Microscopy of MSOME

**Figure 1:**
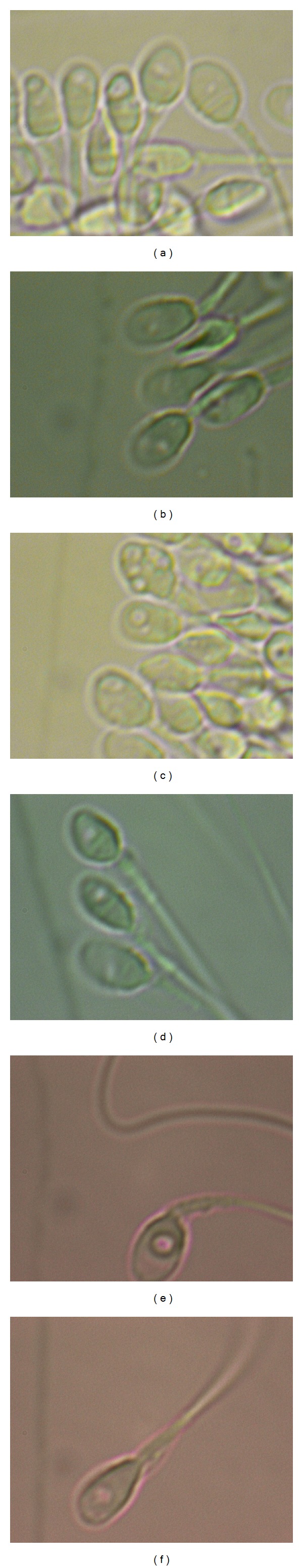
Sperm with head vacuoles in normozoospermic ((a), (c), and (e)) and teratozoospermic ((b), (d), and (f)) samples observed with DIC microscopy (Nomarski) at 6000-time magnification.

In both, the normal and abnormal sperm samples, the vacuoles were located in different parts of the head of spermatozoa. They were seen as surface concavities or lighter translucent areas of various sizes and numbers. In literature, the sperm head vacuoles are described as surface concavities, pocket-like nuclear concavities [8], or nuclear thumb-prints [2] under the light microscope. In our study, the MSOME, using Nomarski DIC microscopy at 6000-time magnification, showed sperm head vacuoles as surface concavities (Figure 1). However, we must take into consideration that Nomarski DIC microscopy results in a pseudo-3D image due to refraction of light passing through the specimen of different thickness and optical density. Therefore, it is possible that nuclear regions of different optical density are not just concavities but also irregular bulges at the cell surface.

#### 3.1.2. Sperm Head Vacuoles, Classical Parameters of Sperm Quality, and Male Factors

Our data show that sperm head vacuoles were present in both the normal and abnormal sperm samples (Figure 1). Interestingly, there was a relatively high proportion (74.6%) of spermatozoa with sperm head vacuoles in the sperm of normal quality, thus indicating that the sperm head vacuoles are also found in normospermic men (Table 1). Although there was a higher proportion of spermatozoa with head vacuoles in men with teratozoospermia than in man with normal sperm quality (90.2% versus 74.6%; *P* < 0.01), as can be seen in Table 1, it is not excluded that sperm head vacuoles may also be related to the sperm pathology in these men. Moreover, in the sperm of men with teratozoospermia, there were a significantly lower proportion of optimal spermatozoa of Class I and a higher proportion of low quality sperm of Classes III and IV than in samples of normal sperm (Table 1)

However, some previous studies reported higher percentages of vacuolated spermatozoa in subfertile men with normal semen parameters, ranging from 97.7% [16] to 98.4% [21]. This discrepancy reflects the fact that the subfertile men with normal semen quality but included into the in vitro fertilization program represent a heterogeneous population of men, from fertile partners of infertile women to men with repeated poor in vitro fertilization outcome in spite of normal semen quality. In addition, there are several different habits, lifestyles, and health conditions (e.g., inflammation and varicocele) that may affect their sperm in terms of head vacuoles. There was a positive correlation between the proportion of “good sperm” (Class I and Class II) in terms of head vacuoles and sperm concentration and sperm morphology (percentage of normal sperm) in men included in this study, as can be seen in Table 2.

Moreover, there was a positive correlation between the proportion of Class I sperm and sperm concentration, motility, and morphology. On the other hand, there was a negative correlation between the proportion of “bad sperm” (Class III and Class IV) in terms of head vacuoles and sperm concentration and morphology (Table 2). In addition, the proportions of Class III and Class IV sperm were negatively correlated with sperm morphology and the proportions of Class IV sperm were also negatively correlated with sperm motility. All of these data indicate that sperm head vacuoles were related to impaired sperm quality, although they were also present in normal sperm samples. This finding is in accordance with some previous studies [21].

Interestingly, there was a significant negative correlation between the male age and concentration of sperm, while we did not observe any correlation between the sperm head vacuoles and male factors such age, height, weight, and body mass index (Table 3).

We also did not find any significant correlation between semen volume or leukocytospermia and sperm head vacuoles. On the other hand, there were more previous studies which showed that some of these male factors may have an impact on sperm quality and formation of head vacuoles [22–24]. One of this studies showed that increased male age and body mass index are related to increased sperm head vacuoles but this may be compensated by higher ejaculation frequency and a shorter period of sexual abstinence in these men [24]. This means that some other still unknown male factors affect the appearance of sperm head vacuoles.

#### 3.1.3. Sperm Head Vacuoles and the Outcome of In Vitro Fertilization

The groups of men included into the study did not differ regarding the mean age of female partners and the number of oocytes per woman, the main female factors that may affect the outcome of in vitro fertilization (Table 4). The pregnancy rates achieved in teratozoospermic and normozoospermic men were 32.5% and 26.8% (see Table 4).

By further inspection, it became clear that the proportion of “good sperm” or “bad sperm” in terms of head vacuoles in semen samples was indicative for a proportion of fertilized oocytes (fertilization rate) after in vitro fertilization procedure but this depended on the method of in vitro fertilization. After classical in vitro fertilization (IVF), there was a significant positive correlation between the percentage of “good sperm” in the semen sample and fertilization rate (Table 5). On the other side, there was a negative correlation between the proportion of “bad sperm” and the fertilization rate, as can be seen in Table 5.

**Figure 2:**
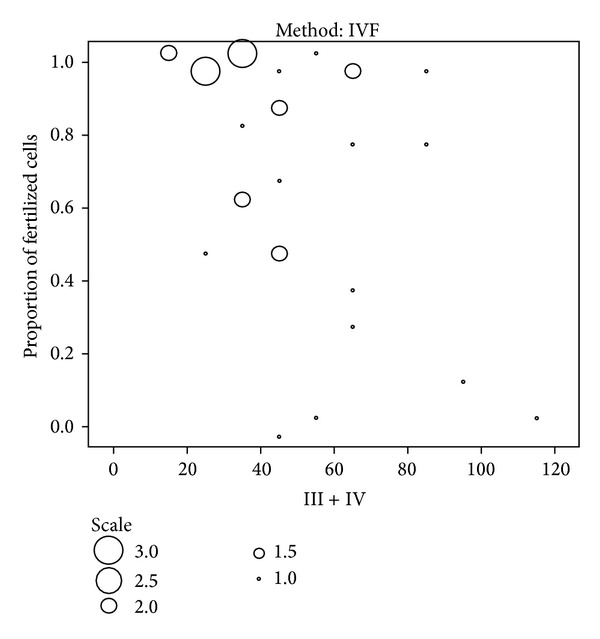
Relation between sperm head vacuoles (proportion of bad sperm: Class II plus Class III) in semen samples and oocyte fertilization rate after classical in vitro fertilization (IVF).

From Figure 2, it can be seen that, in semen samples with more than ~50% of “bad sperm” in terms of sperm head vacuoles, the fertilization rate was decreased. Interestingly, we did not observe any correlation between sperm head vacuoles and fertilization rate after intracytoplasmic injection (ICSI), as found by some previous studies [16]. This indicates that sperm head vacuoles may impair some physiological process of spermatozoa that are needed for classical in vitro fertilization by insemination such as capacitation, acrosome reaction, sperm binding to zona pellucida, or that some more sperm selection is performed by ICSI.

Regarding blastocyst rate, there was a negative correlation (*P* < 0.05) between the percentage of Class III spermatozoa in semen samples and blastocyst rate after classical in vitro fertilization, while we did not observe any other correlations. We did not observe any correlation between sperm head vacuoles and pregnancy rate regardless of the method of in vitro fertilization. There was a positive relation between the pregnancy and number of oocytes retrieved in female partner, as revealed by Mann-Whitney *U* test, but we did not find any relation between pregnancy and sperm head vacuoles. This was further confirmed by multivariate logistic regression respecting female and male factors including sperm head vacuoles.

Most of the previous studies have tried to elucidate the effect of sperm head vacuoles on the outcome of ICSI in men with poor semen quality and some of them indeed confirmed the negative effect [13–15]. The advantage of our study is that we elucidated the effect of sperm head vacuoles not only on the outcome of ICSI in semen samples of poor quality (teratozoospermia) but also on the outcome of classical IVF in normal semen samples (with normal classical parameters of semen quality, including morphology). Our data indicate that the sperm head vacuoles were even more deleterious for the outcome of classical IVF than for ICSI.

### 3.2. Transmission Electron Microscopy (TEM)

**Figure 3:**
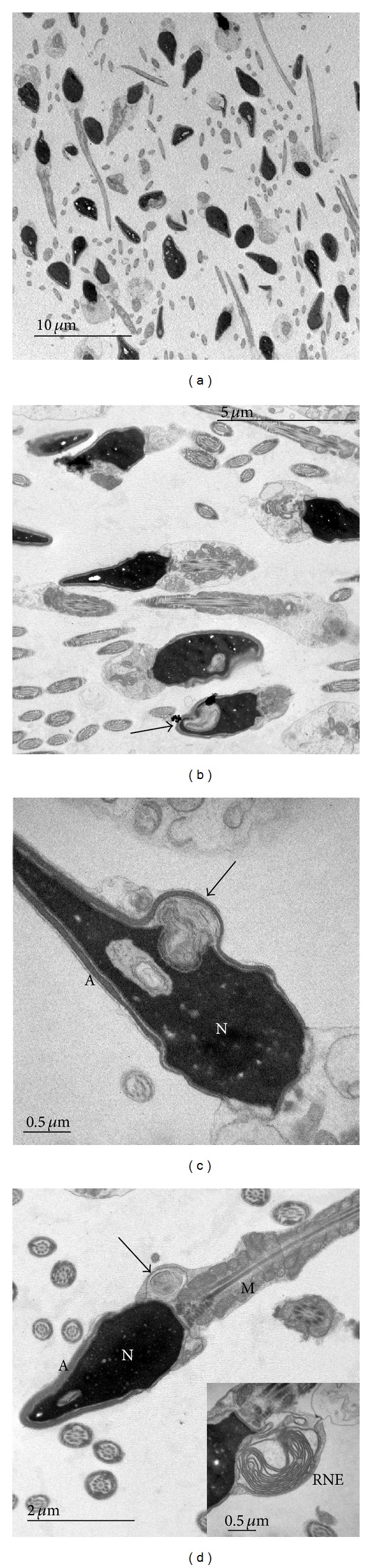
Transmission electron micrographs (TEM) of normozoospermic samples with head vacuoles of various size and position. Arrow indicates vacuoles on (b), (c), and RNE in (d). A: acrosome, N: nucleus, M: mitochondria, and RNE: redundant nuclear envelope.

Similarly, as by MSOME, transmission electron microscopy (TEM) revealed the sperm head vacuoles in normal and abnormal sperm samples. The vacuoles in sperm heads were seen as lighter areas inside the nucleus and were present in almost all spermatozoa in both types of sperm samples (Figures 3(a) and 3(b)). Electron micrographs of sperm head vacuoles are rarely presented in the literature and the interpretation of their ultrastructure is scarce [12, 25]. The description of fine structure of human spermatozoa usually presents large lucent areas in sperm heads as vacuoles and smaller clear areas as nucleoplasm or clear spots [26].

Contrary to light micrographs where vacuoles are seen as surface concavities, in electron micrographs, vacuoles were occasionally observed as bulges at the cell surface (Figure 3(c)). Due to different optical density of vacuoles and condensed chromatin, nuclear indentations packed with membranes appear as surface concavities under light microscopy.

#### 3.2.1. Ultrastructural Features of Sperm Head Vacuoles

**Figure 4:**
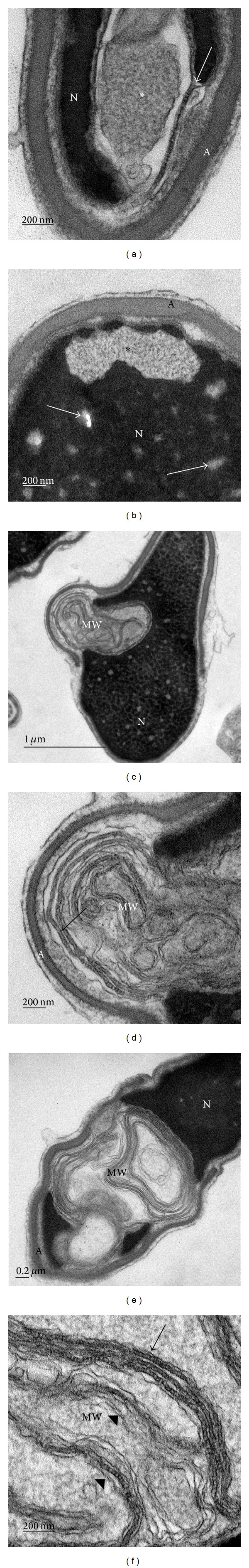
TEM micrographs of sperm head vacuoles of normozoospermia ((a), (c), (d), (e), and (f)) and teratozoospermia (b). (a) Nuclear indentations with membranes enclosing flocculent material (asterisk). Double membrane with molecular septa (arrow) emerging from nuclear envelope. (b) Nucleus with clear spots of noncondensed chromatin (arrow) and large area with flocculent material (asterisk). (c) Membrane whorls (MW) consisting of concentrically arranged membranes. (d) High magnification of membrane whorls of (c). (e) Membrane whorls (MW) occupying a large part of nucleus. (f) High magnification of double membranes with septal complexes (arrow) and thin membranes (arrow head). A: acrosome; N: nucleus.

Electron micrographs of sperm head vacuoles showed nuclear indentations containing stacks of membranes (Figures 4(c), 4(d), and 4(f)). These membranes were organized as concentrically arranged lamellae and are described as membrane whorls (MW), which occasionally bulge from the cell surface. Membrane whorls of various complexities were present in different areas of sperm nucleus and can occupy a large area of nucleus in both morphologically normal and abnormal spermatozoa. In almost all cases, membrane whorls were covered by intact acrosome and plasma membrane (Figures 4(c) and 4(d)). They were mostly composed of tightly packed double membranes with electron dense molecular septa and thinner membranes enclosing flocculent material (Figures 4(d) and 4(f)). Franklin and Fussell [27] observed membrane whorls in apical subacrosomal space of late hamster spermatids which were continuous with the nuclear envelope. According to the authors [27], the membrane whorls appeared larger and were present in greater numbers in the final or maturation phase of spermiogenesis.

**Figure 5:**
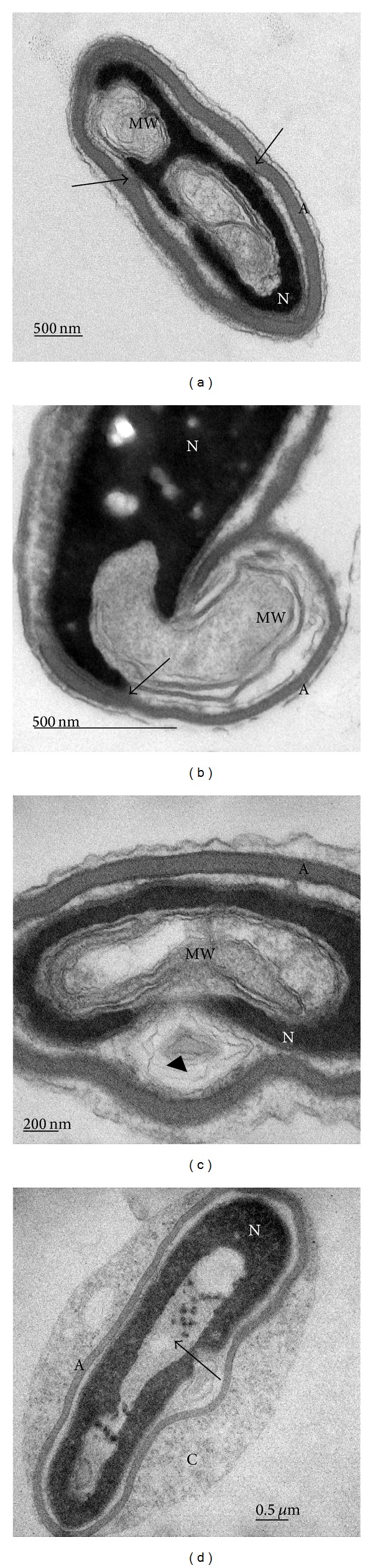
Cross sections of spermatozoa in normozoospermic samples ((a), (b), and (c)) with large nuclear indentations with membrane whorls (MW). Acrosome membrane is connected with nuclear envelope (arrows). (c) Membrane whorls (MW) with additional membranes between nucleus and acrosome (arrowhead). (d) Large part of noncondensed chromatin with granular formation inside the nucleus (arrow) of spermatozoon with abnormal morphology. A: acrosome, N: nucleus, and C: cytoplasm.

Sperm maturation leads to nuclear reorganisation and reduction of its size. From the literature, it is known that nuclear maturation involves reduction of nuclear envelope, a dynamic double membrane that surrounds the nucleus, segregates chromosomes, and regulates nuclear transport through pores [28]. Many details of its transformation are still unclear. Our micrographs of membrane whorls showed continuity of membranes with the nuclear envelope (Figures 5(a) and 5(c)). Membrane structures with septate electron dense molecular complexes are probably produced during the reorganization of the nuclear envelope. These structures were described by several authors as redundant nuclear envelope (RNE) [11, 28, 29]. The RNE is seen in our micrograph of normal spermatozoa (Figure 3(d)).

Different stages of chromatin condensation are seen in our micrographs in all samples. In Figure 5(d), we can see one spermatozoon with abnormal morphology in a process of chromatin condensation which is still surrounded by large amount of cytoplasm. In late spermatogenesis, sperm DNA becomes tightly packed because histones are replaced by protamines [11]. However, condensation defects frequently occur and result in irregularly shaped clear spaces of various sizes in the nucleus [26]. Chromatin condensation failure is known to be associated with the presence of small and large vacuoles [2, 8]. Our micrographs revealed smaller electron lucent areas of noncondensed chromatin and nuclear vacuoles which differ in size and always contain amorphous material or/and membrane whorls. They might be more expressed in spermatozoa of teratozoospermic samples and it may be related to abnormal sperm maturation process and sperm head vacuoles. However, more focused research is required.

Acrosome was fully developed in most spermatozoa with sperm head vacuoles. In some cells, interconnections of inner acrosomal membrane and nuclear envelope were observed (Figures 5(a) and 5(b)). Acrosome morphogenesis is a dynamic process that involves a close interaction between Golgi complex and nuclear envelope of early spermatid. It has been shown that perinuclear theca (PT) is a cytoskeletal structure that covers the sperm nucleus in mammals and has been shown to play important role in normal acrosome formation [30].

As reviewed by Setti et al. [31], some authors report that sperm vacuoles are of acrosomal origin [4] and reflect nonreacted acrosome [5]. A negative relation between the presence of vacuoles and the sperm capacity to undergo the acrosomal reaction is exposed. Our data show that the sperm head vacuoles are unique structures, usually covered by intact acrosome.

**Figure 6:**
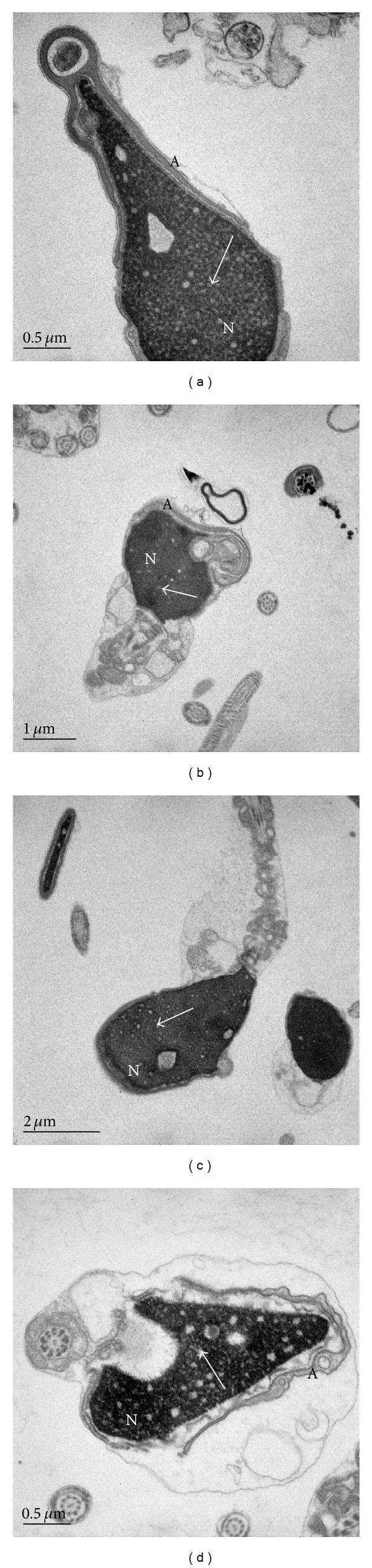
(a), (b), (c), and (d) Spermatozoa from normal sperm samples with abnormal morphology. Chromatin is granular and less condensed with numerous clear spots (white arrows). Acrosome is abnormally formed (a), partly formed (d), and sometimes connected to membrane whorls (b). (c) Bended neck region. A: acrosome; N: nucleus.

Abnormal spermatozoa were present in both types of sperm samples—normal and abnormal. Abnormalities affected different components of the sperm cells, namely, chromatin, acrosome, neck region, mitochondria, and tail. We present several micrographs of abnormal spermatozoa from normozoospermic samples with less condensed chromatin, bended neck, atypical acrosome, and abnormal head shape (Figure 6).

#### 3.2.2. Function of Sperm Head Vacuoles

At present, it is impossible to state what the real function of sperm head vacuoles is. Based on our micrographs of sperm ultrastructure, we suggest that sperm head vacuoles may remove some substance of unknown origin from chromatin. Because they indicate the potential relation to both the sperm maturation process in one side and abnormal chromatin condensation in the other, one may speculate that the sperm head vacuoles may be involved in the removal of histones when replaced by protamines. But it is too early for any conclusion and further research is needed.

## 4. Conclusions

The results of this study indicate that sperm samples of men with teratozoospermia are characterized by increased ratio of sperm head vacuoles, although they are present also in sperm samples of normospermic men. This indicates that they may be related to pathological state. Our micrographs on sperm ultrastructure indicate that sperm head vacuoles are composed of membrane whorls. Variations in chromatin condensation were mostly observed in abnormal sperm samples and it is not excluded that they are related to the abnormal maturation and sperm head vacuoles. Further research combining different methods and transmission electron microscopy is needed to better elucidate this phenomenon.

## Figures and Tables

**Table 1:** Sperm quality in normospermic men and in men with teratozoospermia: classical parameters of sperm quality and sperm head vacuoles.

Sperm quality	Normozoospermia	Teratozoospermia
Number of patients	40	41
Mean concentration of sperm (×10^6^ spz/mL)	95.5 (min. 50–max. 200)	48.4 (min. 2–max. 100)
Mean sperm motility (% of motile spz)	72.0% (min. 50–max. 80)	65.1% (min. 14–max. 80)
Mean sperm morphology (% of normal spz)	35.6% (min. 18–max. 67)	8.0% (min. 2–max. 13)
% of sperm with head vacuoles	74.6%*	90.2%*
% of Class I sperm (no vacuoles)	26.5%*	9.7%*
% of Class II sperm (at most two small vacuoles)	47.1%	44.6%
% of Class III sperm (more than two small vacuoles or one big vacuole)	25.4%**	37.8%**
% of Class IV sperm (big vacuoles)	2.1%*	7.8%*

*Statistically significant difference (*P* < 0.01).

**Statistically significant difference (*P* < 0.05), as revealed by chi-square test.

**Table 2:** Correlation between sperm head vacuoles (proportions of Classes I–IV) sperm with classical parameters of sperm quality (concentration, motility, and morphology).

Correlations			
	Sperm quality		
	Concentration	Motility	Morphology
Spearman's rho			
I plus II			
Correlation coefficient	,233*	,201	,326**
Significance (2-tailed)	,037	,074	,004
*N*	80	80	78
III plus IV			
Correlation coefficient	−,238*	−,208	−,328**
Significance (2-tailed)	,033	,063	,003
*N*	80	80	78
I			
Correlation coefficient	,321**	,222*	,529**
Significance (2-tailed)	,004	,048	,000
*N*	80	80	78
II			
Correlation coefficient	,055	,111	−,091
Significance (2-tailed)	,627	,326	,429
*N*	80	80	78
III			
Correlation coefficient	−,188	−,138	−,275*
Significance (2-tailed)	,095	,222	,015
*N*	80	80	78
IV			
Correlation coefficient	−,217	−,225*	−,267*
Significance (2-tailed)	,054	,044	,018
*N*	80	80	78

*Correlation is significant at the 0.05 level (2-tailed).

**Correlation is significant at the 0.01 level (2-tailed).

**Table 3:** Correlation between male factors (age, height, weight, and body mass index), classical parameters of sperm quality, and sperm head vacuoles.

Correlations				
	Male factors			
	Age	Height	Weight	BMI
Spearman's rho				
I plus II				
Correlation coefficient	−,183	−,009	−,066	−,098
Significance (2-tailed)	,124	,945	,595	,431
*N*	72	67	67	67
III plus IV				
Correlation coefficient	,178	,003	,060	,095
Significance (2-tailed)	,135	,978	,632	,446
*N*	72	67	67	67
I				
Correlation coefficient	−,150	,042	−,022	−,069
Significance (2-tailed)	,209	,733	,862	,578
*N*	72	67	67	67
II				
Correlation coefficient	−,038	−,085	−,071	−,054
Significance (2-tailed)	,748	,496	,570	,663
*N*	72	67	67	67
III				
Correlation coefficient	,137	,045	,086	,095
Significance (2-tailed)	,250	,716	,488	,442
*N*	72	67	67	67
IV				
Correlation coefficient	,036	−,042	−,073	−,037
Significance (2-tailed)	,762	,733	,558	,766
*N*	72	67	67	67
Concentration				
Correlation coefficient	−,246*	−,151	−,100	−,031
Significance (2-tailed)	,039	,225	,425	,807
*N*	71	66	66	66
Motility				
Correlation coefficient	−,138	−,116	,017	,078
Significance (2-tailed)	,250	,355	,890	,535
*N*	71	66	66	66
Morphology				
Correlation coefficient	−,151	−,154	−,108	−,027
Significance (2-tailed)	,215	,226	,397	,832
*N*	69	64	64	64

*Correlation is significant at the 0.05 level (2-tailed).

BMI: body mass index.

**Table 4:** In vitro fertilization outcome (pregnancy rate) according to the sperm quality (normozoospermia and teratozoospermia).

In vitro fertilization outcome	Normozoospermia	Teratozoospermia
Number of patients (cycles)	40	41
Mean number of previous cycles	2.0 (min. 1–max. 5)	1.2 (min. 1–max. 6)
Mean age of female partners (years)	34 (min. 25–max. 43)	34 (min. 25–max. 42)
Mean number of oocytes per woman (cycle)	7.7 (min. 1–max. 24)	8.0 (min. 1–max. 18)
Number of pregnancies	13	11
Pregnancy rate per cycle	32.5%	26.8%

Statistical significance was set at *P* < 0.05.

**Table 5:** Correlation between sperm head vacuoles (“good sperm”: Class I plus Class II; “bad sperm”: Class II plus Class IV) and outcome of in vitro fertilization (fertilization and blastocyst rates) according to the method of in vitro fertilization.

	Correlations		
	Method	I plus II	III plus IV
Spearman's rho	IVF		
	Fertilization rate		
	Correlation coefficient	,358*	−,358*
	Significance (2-tailed)	,041	,041
	*N*	33	33
	Blastocyst rate		
	Correlation coefficient	,261	−,261
	Significance (2-tailed)	,207	,207
	*N*	25	25
	ICSI		
	Fertilization rate		
	Correlation coefficient	−,050	,048
	Significance (2-tailed)	,738	,747
	*N*	48	48
	Blastocyst rate		
	Correlation coefficient	−,041	,042
	Significance (2-tailed)	,809	,805
	*N*	37	37

*Correlation is significant at the 0.05 level (2-tailed).

IVF: classical in vitro fertilization; ICSI: intracytoplasmic sperm injection.
